# Cofactor recycling for co-production of 1,3-propanediol and glutamate by metabolically engineered *Corynebacterium glutamicum*

**DOI:** 10.1038/srep42246

**Published:** 2017-02-08

**Authors:** Jinhai Huang, Yao Wu, Wenjun Wu, Ye Zhang, Dehua Liu, Zhen Chen

**Affiliations:** 1 Department of Chemical Engineering, Tsinghua University, Beijing 100084, China; 2 Tsinghua Innovation Center in Dongguan, Dongguan 523808, China

## Abstract

Production of 1,3-propanediol (1,3-PDO) from glycerol is a promising route toward glycerol biorefinery. However, the yield of 1,3-PDO is limited due to the requirement of NADH regeneration via glycerol oxidation process, which generates large amounts of undesired byproducts. Glutamate fermentation by *Corynebacterium glutamicum* is an important oxidation process generating excess NADH. In this study, we proposed a novel strategy to couple the process of 1,3-PDO synthesis with glutamate production for cofactor regeneration. With the optimization of 1,3-PDO synthesis route, *C. glutamicum* can efficiently convert glycerol into 1,3-PDO with the yield of ~ 1.0 mol/mol glycerol. Co-production of 1,3-PDO and glutamate was also achieved which increased the yield of glutamate by 18% as compared to the control. Since 1,3-PDO and glutamate can be easily separated in downstream process, this study provides a potential green route for coupled production of 1,3-PDO and glutamate to enhance the economic viability of biorefinery process.

1,3-Propanediol (1,3-PDO) is an important platform chemical which is used in a wide range of areas, including the textile industry, solvent, food, lubricants, and medicine^1^,^2^,^3^,^4^. Of particular interest is its use as a monomer for the synthesis of polyethers, polyurethanes and polyesters such as polytrimethylene terephthalate (PTT)^5^. Biological production of 1,3-PDO has received broad interest in recent years. Dupont has paid large efforts to develop a recombinant *Escherichia coli* which can directly utilize glucose for 1,3-PDO production with high titer and yield^1^. This process has been commercialized and considered as a milestone of metabolic engineering. In recent years, the fast development of biofuel industry has generated a large amount of crude glycerol as byproduct^6^,^7^,^8^. The excess of crude glycerol produced in the biofuel industry is leading to a dramatic decrease in glycerol price, making it a waste with a disposal cost for many biodiesel plants^9^,^10^. Production of 1,3-PDO from cheap and abundant crude glycerol is becoming economically competitive to glucose-based process, representing a promising route toward glycerol biorefinery^11^.

Several species of microorganisms, including *Klebsiella*^12^, *Clostridia*^13^, *Enterobacter*^14^, *Citrobacter*^15^, and *Lactobacilli*^16^, can directly convert glycerol into 1,3-PDO via two metabolic steps: the dehydration of glycerol to 3-hydroxypropionaldehyde (3-HPA) by glycerol dehydratase and the reduction of 3-HPA to 1,3-PDO by 1,3-propanediol dehydrogenase (Fig. 1). Since the production of 1,3-PDO is a reduction process, regeneration of NADH via glycerol oxidation is required. Currently established fermentation process can only reach a maximum yield of 0.5–0.6 mol 1,3-PDO/mol glycerol, with about 40–50% of glycerol converted to undesired by-products such as formate, acetate, lactate, and 2,3-butanediol^4^,^5^,^11^. The generation of large amounts of byproducts not only reduce the atom economy but also significantly increase the complexity of downstream process. It is estimated that the downstream process makes more than 50% of the total costs in 1,3-PDO production^17^. To reduce byproduct formation and increase process economic viability, lignocellulosic hydrolysates were recently used as co-substrates for glycerol fermentation, resulting in an 18–28% increase of 1,3-PDO yield^18^. Co-production of 1,3-PDO with 3-hydroxypropionic acid (3-HP), an oxidation product of glycerol, for NADH recycling has also been achieved in *K. pneumoniae* with an overall yield of 0.66 mol 1,3-PDO plus 3-HP per mol glycerol^19^.

*Corynebacterium glutamicum* is a gram-positive bacterium which has been widely used for amino acids production in industry^20^,^21^,^22^,^23^,^24^. Recently, applications of this microorganism have been extended for the production of other chemicals, such as isobutanol^25^, cadaverine^26^, and ethylene glycol^27^. Glutamate fermentation by *C. glutamicum* is a well-established industrial process which produces about 2.5 million tons of glutamate per year^28^. During glutamate fermentation, about 3 mol NADH/mol glutamate is produced, which need to be oxidized via oxidative phosphorylation^28^,^29^. The oxidation of excess NADH via oxidative phosphorylation may reduce the yield of glutamate. It was previously reported that disruption of oxidative phosphorylation via the deletion of H^+^-ATPase significantly enhanced glutamate production^30^.

Based on these observation, herein, we propose a novel process to couple the production of 1,3-PDO with glutamate fermentation in *C. glutamicum* for efficient cofactor regeneration (Fig. 1). With the introduction of 1,3-PDO synthesis pathway in *C. glutamicum*, the NADH generated during glutamate fermentation could be recycled for 1,3-PDO production. Theoretically, 1,3-PDO and glutamate can be coproduced with the maximum yield of 1.0 mol PDO/mol glycerol and 1.0 mol glutamate/mol glucose. The produced glutamate and 1,3-PDO can be easily purified via classical separation processes such as crystallization and distillation (Fig. 1). Thus, this process may be integrated into current glutamate production line, increasing the economic viability of glutamate industry. In this study, we first introduced the heterologous 1,3-PDO synthesis pathway in *C. glutamicum*. The production efficiency of 1,3-PDO was further improved via pathway and culture optimization. We showed that co-production of glutamate and 1,3-PDO in one fermentation was possible and the yield of glutamate could be increased in the coupled process. This is also the first report of 1,3-PDO production by industrial important strain *C. glutamicum*.

## Results

### Construction of 1,3-propanediol synthesis pathway in *C. glutamicum*

**Figure 2:**
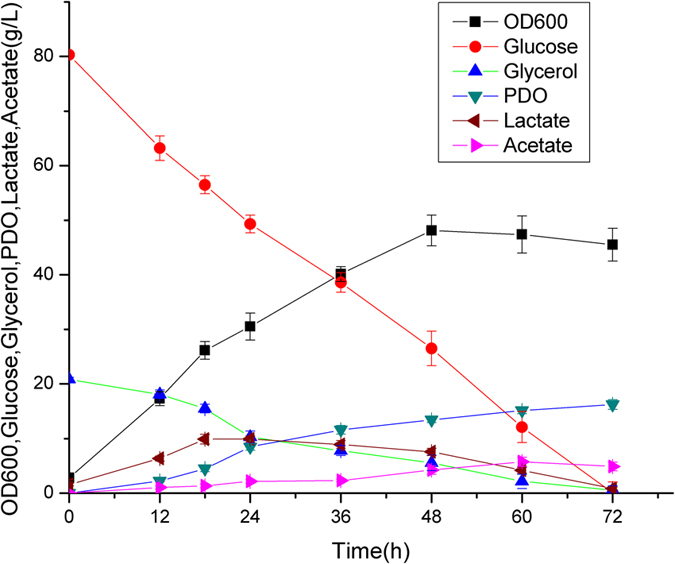
Cell growth, substrates consumption and products formation of *Corynebacterium glutamicum* PT01 under aerobic condition. The fermentation was carried out in aerobic condition (500 ml baffled flasks containing 50 ml modified LPG2 medium at 30 °C and 200 rpm).

There is no glycerol assimilation pathway in *C. glutamicum.* Although *C. glutamicum* possesses a potential glycerol kinase (encoded by *glpK*, cgp_3198)^10^, it cannot utilize glycerol as sole carbon source. To efficiently produce 1,3-PDO, we first started by heterologous expression of 1,3-PDO synthesis pathway in *C. glutamicum* MB001. The *pduCDEGH* gene from *K. penumoniae*, encoding diol dehydratase and its activator, was co-expressed with *dhaT* gene encoding 1,3-PDO dehydrogenase in plasmid pEC-K18mob2 under the control of constitutive lac promoter. The recombinant strain PT01 still cannot utilize glycerol as carbon source, indicating that there is no active glycerol oxidation pathway in *C. glutamicum*. When cultured in LPG2 medium using glucose and glycerol as co-substrates, strain PT01 can grow and simultaneously utilize glucose and glycerol (Fig. 2). 16.2 g/L of 1,3-PDO was produced from 20.3 g/L of glycerol, with the yield of ~1.0 mol PDO/mol glycerol, suggesting that almost all of glycerol is converted into 1,3-PDO. This was consistent with the reported that *C. glutamicum* cannot oxidize glycerol due to the extremely low activity of glycerol kinase and glycerol 3-phosphate dehydrogenase^10^. Therefore, glucose catabolism is utilized for cell growth and regeneration of NADH for 1,3-PDO production. Glucose is mainly converted into CO_2_ via TCA cycle under aerobic condition in LPG2 medium. Besides CO_2_, accumulation of acetate (~5.7 g/L) were also observed during the fermentation (Fig. 2).

### Pathway optimization to improve strain performance

**Figure 3:**
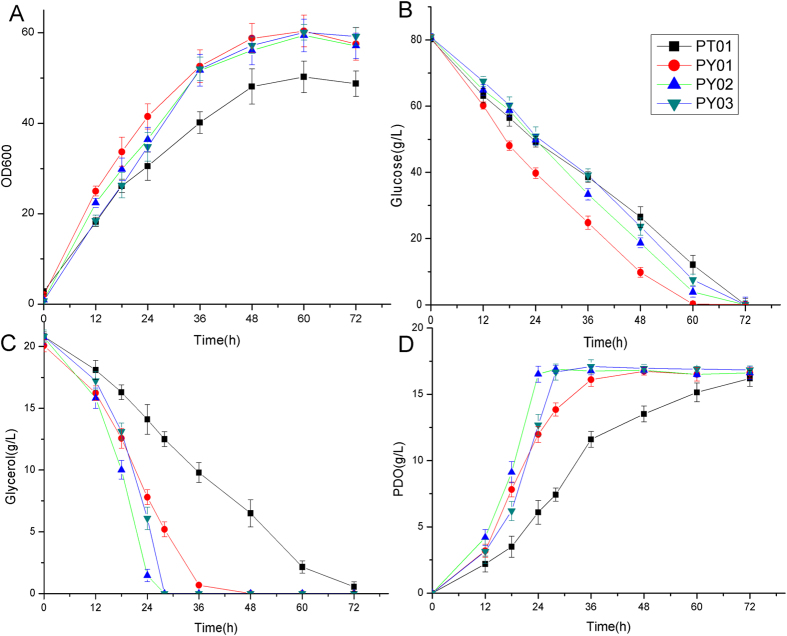
Pathway optimization for improved 1,3-PDO production: (**A**) cell growth; (**B**) glucose consumption; (**C**) glycerol consumption; (**D**) 1,3-PDO production. The fermentations were carried out in aerobic condition (500 ml baffled flasks containing 50 ml modified LPG2 medium at 30 °C and 200 rpm).

**Figure 4:**
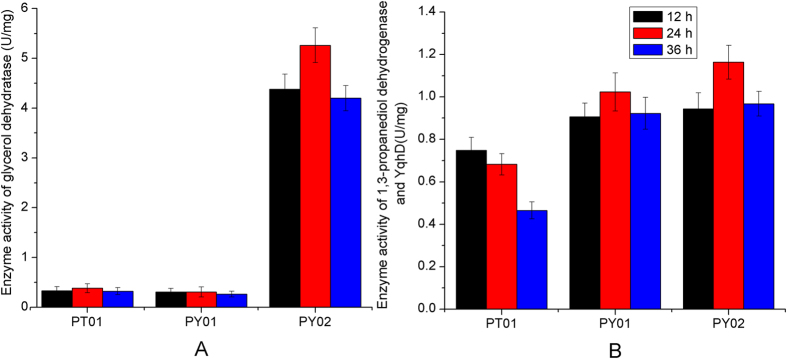
Enzyme activities of glycerol dehydratase (**A**) and 1,3-propanediol dehydrogenases (**B**) of strain PT01, PY01, and pY02. The culture was carried out in aerobic condition (500 ml baffled flasks containing 50 ml modified LPG2 medium at 30 °C and 200 rpm).

Although strain PT01 can produce 1,3-PDO with high yield, the glycerol consumption rate (0.28 g/L/h) and 1,3-PDO production rate (0.23 g/L/h) were relatively low. To improve the production rate of 1,3-PDO, we attempted to further optimize 1,3-PDO synthesis pathway. 1,3-PDO dehydrogenase is the terminal enzyme of 1,3-PDO synthesis pathway which catalyzes the reduction of 3-HPA. There are two enzymes which have been reported to be able to catalyze this reaction: the NADH-dependent alcohol dehydrogenase encoded by *dhaT* gene and NADPH-dependent alcohol dehydrogenase encoded by *yqhD* gene^1^. We substituted the *dhaT* gene in the plasmid of pEC-dhaT-pdu by the *yqhD* gene, constructing the recombinant plasmid of pEC-yqhD-pdu (Supplementary Table 2). The resulting strain PY01 showed significantly improved glycerol consumption rate (0.54 g/L/h vs 0.28 g/L/h) and 1,3-PDO production rate (0.45 g/L/h vs 0.23 g/L/h) (Fig. 3). The yield of 1,3-PDO was not affected (~1.0 mol PDO/mol glycerol). Interestingly, the cell growth and glucose consumption rate were also improved. For the aerobic production of 1,3-PDO in *E. coli*, YqhD was shown to be more effective than DhaT^1^. Under aerobic condition, YqhD can efficiently utilize NADPH as cofactor to catalyze the reduction of 3-HPA. The activity of YqhD for reductive reaction is about 50 times higher than oxidative reaction^31^,^32^,^33^. Contrarily, DhaT utilizes NADH as cofactor and has a high activity for oxidative reaction (~25% of activity for 3-HPA reduction)^34^, which may result in lower efficiency for 1,3-PDO production. Enzyme assay showed that the activity of YqhD in PY01 was significantly higher than DhaT in PT01 especially in the later phase of fermentation (Fig. 4), suggesting that YqhD is also more effective for 1,3-PDO production in *C. glutamicum* in aerobic condition.

Since glycerol dehydratase controls the entry of glycerol into 1,3-PDO synthesis pathway, we attempted to enhance the activity of glycerol dehydratase by inserting a strong promoter H36^35^ in front of *pduCDEGH* (plasmid pEC-yqhD-H36-pdu, Supplementary Table 2). The activity of glycerol dehydratase of the resulting strain PY02 was increased by ~13 fold as compared to strain PY01 (Fig. 4). The activities of 1,3-PDO dehydrogenase of the two strains were comparable (Fig. 4). The glycerol consumption rate and 1,3-PDO production rate were also improved by 50.3% and 48.2%, indicating that glycerol dehydratase is one of the limiting factors for 1,3-PDO production (Fig. 3). Interestingly, the consumption of glucose during 1,3-PDO synthesis (0–36 h) was reduced by 25.4%, suggesting that the oxidation of glucose is more effectively coupled with 1,3-PDO production than strain PY01 since more NADH generated from glucose oxidation was utilized for 1,3-PDO synthesis.

The glycerol facilitator encoded by *glpF* gene of *E. coli* is a transporter of glycerol which has been previously implemented in *C. glutamicum* to improve the assimilation of glycerol^10^. To examine its effect for 1,3-PDO production, the *glpF* gene was inserted into plasmid pEC-yqhD-H36-pdu, giving plasmid pEC-yqhD-glpF-H36-pdu (Supplementary Table 2). The resulting strain PY03 showed similar fermentation profiles as strain PY02 (Fig. 3), suggesting that glycerol transport is not a limiting factor under the tested condition. Glycerol transported by unspecific transporters or passive diffusion was enough under the tested condition.

### Effect of culture conditions for 1,3-propanediol production

**Figure 5:**
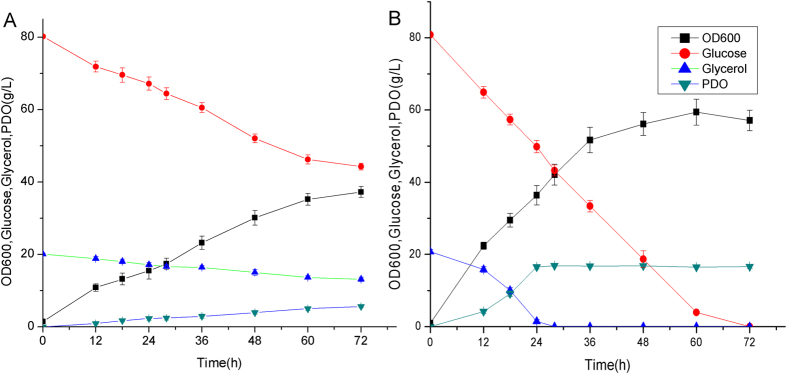
Effect of aeration for 1,3-PDO production by *Corynebacterium glutamicum* PY02. (**A**) Fermentation profile under micro-aerobic condition. The micro-aerobic culture was carried out in 500 ml baffled flasks containing 100 ml modified LPG2 medium at 30 °C and 100 rpm. (**B**) Fermentation profile under aerobic condition. The aerobic culture was carried out in 500 ml baffled flasks containing 50 ml modified LPG2 medium at 30 °C and 200 rpm.

After pathway optimization, we attempted to examine the effect of culture conditions for 1,3-PDO production. Since strain PY02 showed the highest glycerol consumption rate and 1,3-PDO production rate, it was selected for further optimization. Since the activity of glycerol dehydratase is affected by oxygen^36^, we first examined the effect of aeration for 1,3-PDO production. Since *C. glutamicum* cannot grow in anaerobic condition, two conditions were examined: aerobic condition and micro-aerobic condition. For aerobic condition, the cultivation was performed in 500 ml baffled flasks containing 50 ml modified LPG2 medium (20 g/L of glycerol) at 200 rpm. For micro-aerobic condition, the cultivation was performed in 500 ml baffled flasks containing 100 ml modified LPG2 medium (20 g/L of glycerol) at 100 rpm. As shown in Fig. 5, the glycerol consumption and 1,3-PDO production were significantly reduced under micro-aerobic condition, indicating that aerobic condition is beneficial for 1,3-PDO production in *C. glutamicum*. The specific activities of glycerol dehydratase under micro-aerobic condition and aerobic condition were comparable (5.34 ± 0.17 U/mg vs 4.23 ± 0.15 U/mg). However, since the cell grew much faster under aerobic condition, the higher biomass may contribute to the higher glycerol consumption rate.

**Figure 6:**
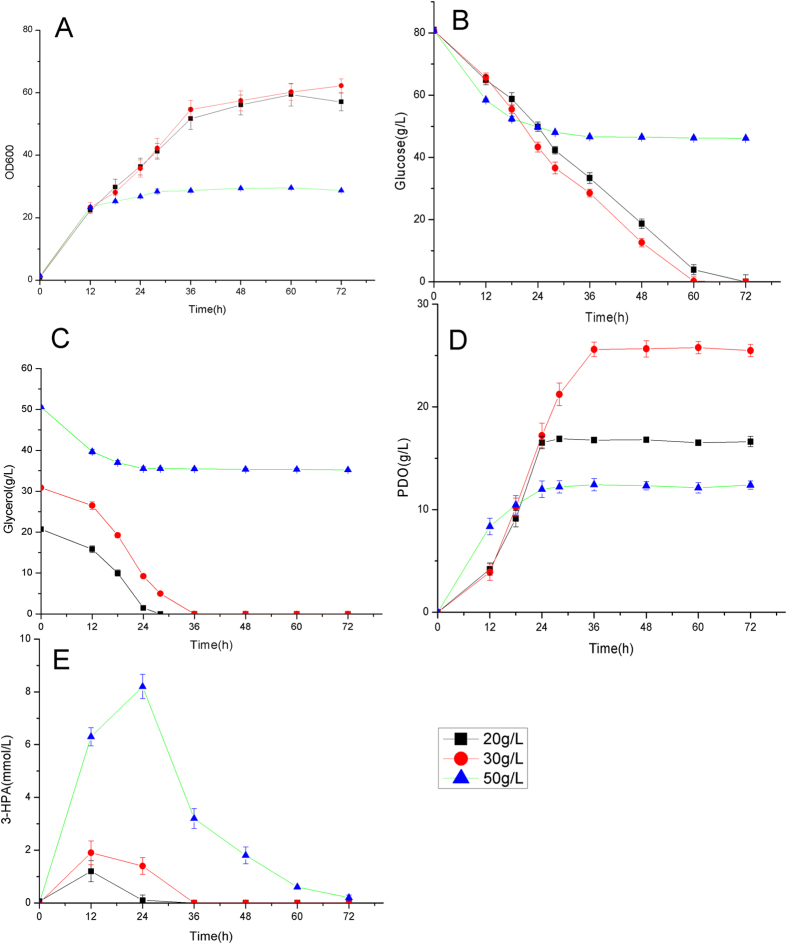
Effect of initial glycerol concentration for 1,3-PDO production by *Corynebaterium glutamicum* PY03. (**A**) cell growth; (**B**) glucose consumption; (**C**) glycerol consumption; (**D**) 1,3-PDO formation; (**E**) 3-hydroxypropionaldehyde (3-HPA) accumulation. The aerobic culture was carried out in 500 ml baffled flasks containing 50 ml modified LPG2 medium at 30 °C and 200 rpm.

Initial concentration of glycerol was reported to be an important factor affecting the production of 1,3-PDO by *K. pneumoniae*^3^,^37^,^38^,^39^,^40^,^41^. We examined three different initial concentrations of glycerol (20 g/L, 30 g/L and 50 g/L) with the same glucose concentration (80 g/L) for the culture of *C. glutamicum* PY02. With the initial concentration of 20 g/L or 30 g/L, glycerol can be completely consumed and the yield of 1,3-PDO is close to 1.0 mol/mol glycerol (Fig. 6). With the initial concentration of 50 g/L, glycerol cannot be completely consumed and cell growth stopped after 24 h. A high accumulation of 3-HPA (8.2 mmol/L) was observed at 24 h (Fig. 6). High concentration of 3-HPA was reported to be very toxic to cell, resulting in the abnormal cessation of fermentation^3^,^37^,^38^,^39^. Thus, it is important to keep the low initial concentration of glycerol.

### Cofactor coupling for simultaneous production of 1,3-propanediol and glutamate

**Figure 7:**
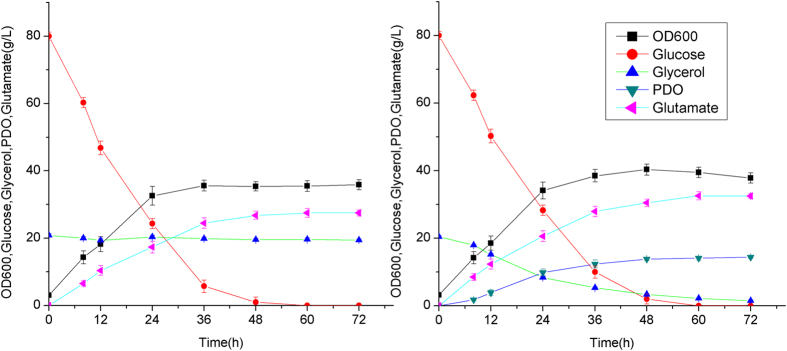
Co-production of 1,3-PDO and glutamate by *C. glutamicum* OD01 and PY04. (**A**) Fermentation profile of strain OD01; (**B**) Fermentation profile of strain PY04. The aerobic culture was carried out in 500 ml baffled flasks containing 50 ml modified LPG2 medium at 30 °C and 200 rpm. The pH was adjusted to 7.0 every 4 h by adding 5 M NaOH.

**Figure 8:**
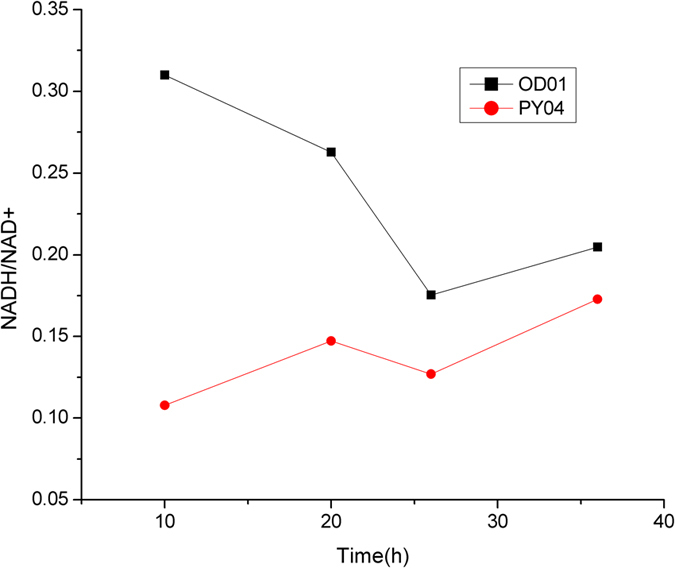
Intracellular concentration of NADH/NAD of strain OD01 and PY04. The aerobic culture was carried out in 500 ml baffled flasks containing 50 ml modified LPG2 medium at 30 °C and 200 rpm. The pH was adjusted to 7.0 every 4 h by adding 5 M NaOH.

In the previous sessions, 1,3-PDO production was not coupled with glutamate production. Glucose was only used for cell growth and generation of reducing equivalent. Glucose was mainly catabolized to CO_2_ and other byproducts such as acetate. To couple the production of 1,3-PDO with glutamate, the pEC-yqhD-H36-pdu plasmid was transformed into *C. glutamicum* OD01, generating strain PY04. *C. glutamicum* OD01 is a modified MB001 strain with the deletion of *odhA* gene, which can accumulate high amount of glutamate without induction^29^. Strain PY04 and OD01 were both cultured in LPG2 medium with 80 g/L of glucose and 20 g/L of glycerol under aerobic condition. As shown in Fig. 7, strain PY04 can consume both glucose and glycerol for glutamate and 1,3-PDO production. 14.4 g/L of 1,3-PDO was produced with the yield of 0.89 mol 1,3-PDO/mol glycerol. Strain PY04 also produced 32.5 g/L of glutamate with the yield of 0.50 mol/mol glucose. The yield of glutamate by strain PY04 was increased by 18.2% as compared to the control strain OD01, suggesting that 1,3-PDO synthesis is beneficial for glutamate production. The intracellular redox state of engineered strains was investigated during the cultivation. As shown in Fig. 8, the ratio of NADH/NAD in strain PY04 was significantly lower than that of strain OD01, indicating that NADH generated from glutamate synthesis was utilized for 1,3-PDO production. The amount of NADH used for 1,3-PDO formation accounted for ~28.4% of NADH generated during glutamate synthesis.

## Discussion

Microbial production of 1,3-PDO from renewable resources has been widely investigated in recent years^4^,^5^,^11^. Due to the reduction of glycerol price^9^,^10^, direct conversion of glycerol into 1,3-PDO is becoming an appealing approach for 1,3-PDO production. Among the natural 1,3-PDO producers, *K. pneumoniae* was mostly studied due to its high productivity^12^,^42^. However, the industrial application of *K. pneumoniae* is limited since it is an opportunistic pathogen. Thus, development of a safe 1,3-PDO producer is highly desirable. *E. coli* as a model organism has been engineered for 1,3-PDO production from glycerol^43^. In this study, *C. glutamicum*, a GRAS (generally regarded as safe) strain, was engineered for the first time to produce 1,3-PDO from glycerol. *C. glutamicum* is a widely used industrial workhorse which is currently utilized for production of million tons of amino acids including glutamate and lysine^20^,^21^,^22^,^23^,^24^. *C. glutamicum* cannot oxidize glycerol^10^, thus providing a good opportunity to build a strain with high yield of 1,3-PDO. With the introduction of 1,3-PDO synthesis pathway, the engineered *C. glutamicum* strains can convert almost all of glycerol into 1,3-PDO (~1.0 mol PDO/mol glycerol). The conversion of glycerol to 1,3-PDO is only catalyzed by two enzymes. However, the balance of enzyme activities between the two enzymes is very important. Increasing the expression of diol dehydratase accelerated glycerol consumption rate and 1,3-PDO formation rate. However, the toxic intermediate 3-HPA was accumulated at high initial glycerol concentration which caused the abnormal cessation of fermentation. 3-HPA accumulation was also observed in *Enterobacterial agglomerans* and *K. pneumoniae*^3^,^37^,^38^,^39^. Imbalance of activities of diol dehydratase and 1,3-PDO dehydrogenase were also observed during 3-HPA accumulation^3^,^37^,^38^,^39^. Two potential reasons may cause this phenomenon. First, high glycerol concentration could enhance the rate of glycerol dehydration since the Km value of glycerol for diol dehydratase was reported to be relatively high (about 16 mM)^44^. Second, high ratio of NAD/NADH was observed at high glycerol concentration which may also result in the lower rate of 3-HPA reduction^39^. Lim *et al*. reported that application of UTR (untranslated region) engineering to precisely control the expression of glycerol dehydrogenase for optimal metabolic balance could significantly enhance the production of 3-HP^45^. Similar strategy could also be applied for improving the production of 1,3-PDO.

When glucose was utilized as carbon source for cell growth and NADH generation, about ~50 g/L of glucose was consumed for the conversion of 30 g/L of glycerol (Fig. 5). To increase the process economy, we propose that glucose can be oxidized into glutamate, another important industrial product. The cofactor can be recycled for the co-production of 1,3-PDO and glutamate. It was shown that this coupled process increased the yield of glutamate (Fig. 7). Although the titers of 1,3-PDO and glutamate production were not very high, they may be further improved by combining system metabolic engineering strategies and process optimization. Since glutamate and 1,3-PDO can be easily separated in downstream processes, this study provides a promising alternative for 1,3-PDO and glutamate production with high atom economy. The same strategy may be applied for the production of other oxidized and reduced products.

## Methods

### Bacterial strains and plasmids

Strains and plasmids used in this study are listed in Table 1. *E. coli* DH5α was used for routine cloning procedures. *C. glutamicum* MB001, a prophage-free strain derived from *C. glutamicum* ATCC 13032, was used as a background strain^46^. *C. glutamicum* OD01, which can produce glutamate without induction, is derived from strain MB001 with the deletion of *odhA* gene encoding a subunit of α-ketoglutarate dehydrogenase^29^. pEC-K18mob2 is a *E. coli/C. glutamicum* shuttle vector used for gene overexpression^47^.

### Plasmids and strains construction

The *yqhD* gene encoding alcohol dehydrogenase and *pduCDEGH* gene encoding diol dehydratase and its activator were amplified from the genome of *E. coli* K12 and *K. pneumoniae* DSM 2026 using primers 11-F/11-R and 12-F/12-R. The two fragments were inserted into the restriction site of EcoRI/XbaI of pEC-K18mob2 by Gibson Assembly Master Kit (NEB)^48^, giving recombinant plasmid pEC-yqhD-pdu. To construct plasmid pEC-dhaT-pdu, the *dhaT* gene encoding 1,3-PDO dehydrogenase was PCR amplified from the genome of *K. pneumoniae* DSM 2026 using primers 13-F/13-R. The backbone of pEC-yqhD-pdu was also PCR amplified using primers 14-F/14-R. The *dhaT* fragment and pEC-yqhD-pdu backbone were assembled into plasmid pEC-dhaT-pdu by Gibson Assembly Master Kit (NEB). To insert an artificial constitutive promoter H36^35^ in front of *pduCDEGH*, the H36 promoter was amplified from plasmid pEC-H36^27^ using primers 15-F/15-R. The backbone of pEC-yqhD-pdu was also PCR amplified using primers 16-F/16-R. The two fragments were assembled by Gibson Assembly Master Kit (NEB), giving plasmid pEC-yqhD-H36-pdu. To construct plasmid pEC-glpF-yqhD-H36-pdu, the *glpF* gene encoding glycerol facilitator was PCR amplified from the genome of *K. pneumoniae* DSM 2026 using primers 17-F/17-R. The fragment was inserted into the restriction site of EcoRI of pEC-yqhD-H36-pdu by Gibson Assembly Master Kit (NEB).

The recombinant plasmids were transformed into *C. glutamicum* strains MB001 and OD01 using electroporation as described by van der Rest *et al*.^49^. The correct recombinants were verified by colony PCR and sequence analysis. All of the primers used in this study were listed in Supplementary Table 1.

### Culture condition

Lysogenic broth (LB) medium was used for the routine culture of *C. glutamicum* strains. Production of 1,3-PDO by *C. glutamicum* was performed in 500 ml baffled flasks. For aerobic cultivation, the culture was carried out in 500 ml baffled flasks containing 50 ml modified LPG2 medium at 30 °C and 200 rpm. For micro-aerobic cultivation, the culture was carried out in 500 ml baffled flasks containing 100 ml modified LPG2 medium at 30 °C and 100 rpm. The modified LPG2 medium consists (per liter): 80 g glucose, 10–50 g glycerol, 10 g corn steep liquor, 20 g (NH_4_)_2_SO_4_, 4.5 g urea, 0.5 g MgSO_4_·7H_2_O, 0.5 g KH_2_PO_4_, 10 mg FeSO_4_·7H_2_O, 10 mg MnSO_4_·H_2_O, 0.2 mg biotin, 5 mg thiamine-HCl, 50 μM of vitamin B12, and 30 g CaCO_3_. When appropriate, the medium was supplemented with 25 μg/ml kanamycin. For the coproduction of 1,3-PDO and glutamate, the pH decreased dramatically during the cultivation. Thus, we manually adjusted the pH to 7.0 every 4 h by adding 5 M NaOH. All experiments were repeated for three times.

### Enzyme assays

The activity of glycerol dehydratase was assayed by the 3-methyl-2-benzothiazolinone hydrazone (MBTH) method as described by Toraya *et al*.^50^. The reaction (in a total volume of 1 mL) contains 0.05 M KCl, 0.2 M 1,2-propanediol, 15 μM coenzyme B12, and 0.035 M potassium phosphate buffer solution (pH 7.0). The assay was started by the addition of cell extract and incubated at 37 °C. After 10 min incubation, the reaction was stopped by adding 1 mL of 0.1 M potassium citrate buffer (pH 3.6). For developing the color, 0.5 mL of 0.1% MBTH solution was added and the mixture was incubated again for 15 min at 37 °C. The color was detected at 305 nm.

The activity of 1,3-PDO dehydrogenase (DhaT and YqhD) was assayed using the reverse reaction^34^,^51^. The reaction mixture contained 30 mM ammonium sulfate, 0.1 M 1,3-PDO, 2 mM NAD (or NADP for YqhD), and 0.1 M potassium carbonate buffer solution (pH 9.0). The reaction was started by adding cell extract and the increase of NADH at 340 nm was monitored with a spectrophotometer.

All of the enzyme assays were repeated for three times.

### Analytical method

The cell density was determined by monitoring the absorbance at 600 nm using a spectrophotometer. Glucose, glycerol, 1,3-PDO and other organic acids were quantified by using High performance liquid chromatography (HPLC) equipped with an Aminex HPX-87H Column (300 × 7.8 mm) using 0.005 M H_2_SO_4_ as the mobile phase with a flow rate of 0.6 mL/min, and detection via refractive index or UV absorption at 210 nm^52^. Glutamate concentration was determined by a glutamate biosensor (SBA-40C, Shandong Science Academy, Jinan, China)^53^. The concentration of 3-HPA was measured according to the method described by Krauter *et al*.^54^. A mixture of 200 μL sample, 150 μL tryptophan-reagent (10 mM DL-tryptophan, 50 mM HCl) and 600 μL HCl (37%) was mixed, incubated at 37 °C for 20 min and measured at 650 nm. 3-HPA was chemically synthesized from acrolein by the method reported by Hall and Stern^55^. NADH and NAD concentrations were determined using NAD/NADH assay kit (Sigma, USA) following the manufacturer’s protocol.

## Additional Information

**How to cite this article**: Huang, J. *et al*. Cofactor recycling for coproduction of 1,3-propanediol and glutamate by metabolically engineered *Corynebacterium glutamicum. Sci. Rep.* **7**, 42246; doi: 10.1038/srep42246 (2017).

**Publisher's note:** Springer Nature remains neutral with regard to jurisdictional claims in published maps and institutional affiliations.

## Supplementary Material

Supplementary Table 1 and Table 2

## Figures and Tables

**Figure 1:**
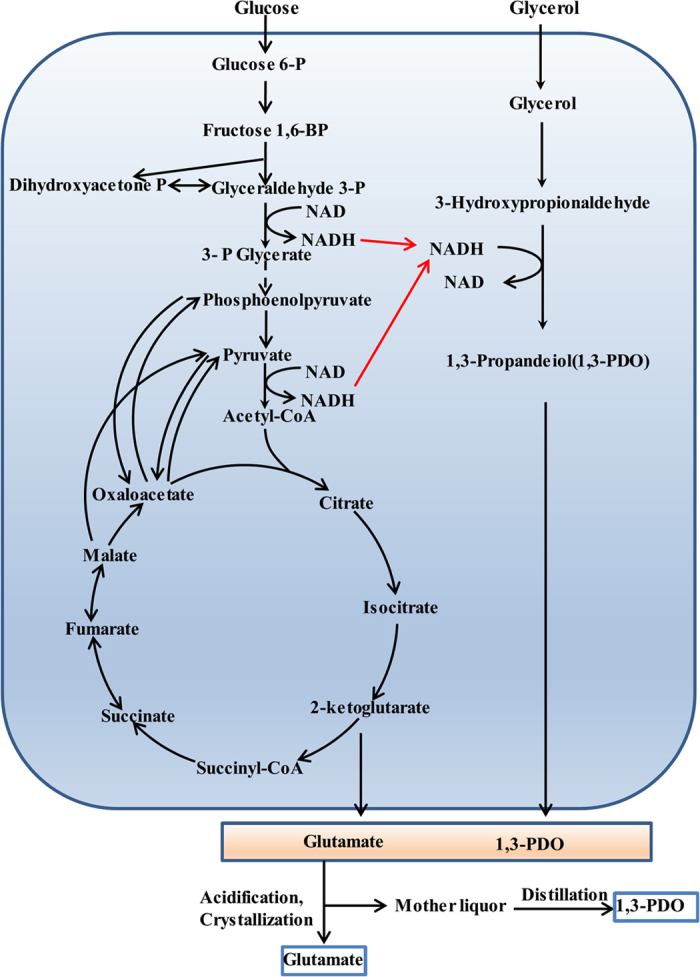
Co-production of 1,3-PDO with glutamate by *Corynebacterium glutamicum*. The reducing equivalents generated during glutamate fermentation can be recycled for 1,3-PDO production. 1,3-PDO and glutamate can be easily separated via classical separation processes such as crystallization and distillation.

**Table 1:** Strains and plasmids used for this study.

Strain or plasmid	Description	Reference
MB001	Prohage-free variant of *Corynebacterium glutamicum* ATCC 13032	13
OD01	MB001 with the deletion of *odhA* gene, which can produce glutamate without induction	Lab collection
PT01	MB001/pEC*-*dhaT-pduCDEGH	This study
PY01	MB001/pEC*-*yqhD-pduCDEGH	This study
PY02	MB001/pEC-yqhD-H36-pduCDEGH	This study
PY03	MB001/pEC-yqhD-glpF-H36-pduCDEGH	This study
PY04	OD01/pEC-yqhD-H36-pduCDEGH	This study
Plasmids		
pEC-K18mob2	*E. coli/C. glutamicum* shuttle vector, Km^r^, ori pGA1	15
pEC*-*dhaT-pduCDEGH	pEC-K18mob2 containing *dhaT* and *pduCDEGH* gene from *K. pneumoniae*	This study
pEC*-*yqhD-pduCDEGH	pEC-K18mob2 containing yqhD gene from *E. coli* and *pduCDEGH* gene from *K. pneumoniae*	This study
pEC-yqhD-H36-pduCDEGH	pEC*-*yqhD-pduCDEGH with the insertion of H36 promoter in front of *pduCDEGH*	This study
pEC-yqhD-glpF-H36-pduCDEGH	pEC*-*yqhD-H36-pduCDEGH with the insertion of glpF gene between yqhD and H36	This study
