# Supplementary material for: Cofactor recycling for co-production of 1,3-propanediol and glutamate by metabolically engineered Corynebacterium glutamicum
Source: Sci Rep. 2017 Feb 8;7:42246. doi: 10.1038/srep42246 (PMC5296756; doi:10.1038/srep42246)
Supplement: Supplementary Table 1 and Table 2 [file srep42246-s1.pdf]

Cofactor recycling for co-production of 1,3-propanediol and glutamate by metabolically engineered *Corynebacterium glutamicum*

Jinhai Huang[1], Yao Wu[1], Wenjun Wu[1], Ye Zhang[1], Dehua Liu[1,2] and Zhen Chen[1,2]*

1. Department of Chemical Engineering, Tsinghua University, Beijing 100084, China
2. Tsinghua Innovation Center in Dongguan, Dongguan 523808, China

**Corresponding author*: Zhen Chen E-mail: zhenchen2013@mail.tsinghua.edu.cn

Tel: +86-10-62772130. Fax: +86-10-62792128

Supplementary Table 1：Primers used for the construction of *C. glutamicum* mutants

Name	Sequence	Purpose
11-F	CTATGACATGATTACGAATTAAGGAGATAT ACCATGAACAACTTTAATCTGCAC	To amplify gene of yqhD
11-R	ATATCTCCTTTTAGCGGGCGGCTTCG	
12-F	CGCCCGCTAAAAGGAGATATACCATGAGAT CGAAAAGATTTGAAG	To amplify gene of pduCDEGH
12-R	TGCCTGCAGGTCGACTCTAGTTAAGCATGG CG	
13-F	ACGAATTAAGGAGATATACCATGAGCTATC GTATGTTTGATTATCTGGTG	To amplify gene of dhaT
13-R	ATCTCATGGTATATCTCCTTTCAGAATGCCT GGCGGAAAA	
14-F	AAGGAGATATACCATGAGATCGAAAAGAT TTGAA	To amplify the backbone of pEC-yqhD-pdu
14-R	GGTATATCTCCTTAATTCGTAATCATGTCAT AGCTGT	
15-F	TATACGAAGCCGCCCGCTAAGAATTCCAAA AGCTGGGTACCTCTATCTG	To amplify the H36 promoter from plasmid pEC-H36
15-R	TCAAATCTTTTCGATCTCATGGATCCCATGC TACTCCTACC	
16-F	ATGAGATCGAAAAGATTTGAAGCACTG	To amplify the backbone of pEC-yqhD-pdu
16-R	TTAGCGGGCGGCTTCGTATAT	
17-F	TATATACGAAGCCGCCCGCTAAGATTTCAG GATGCCGATAATGAACCAG	To amplify the gene of glpF
17-R	TAGAGGTACCCAGCTTTTGGTTACTCATCG ATACCGCATTCGCAC	

Supplementary Table 2. Operon structure in different expression vectors.

Strains	Plasmid	Operon
**PT01**	pEC-dhaT-pdu	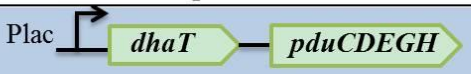
**PY01**	pEC-yqhD-pdu	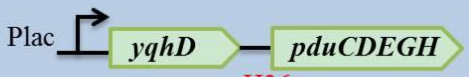
**PY02**	pEC-yqhD-H36-pdu	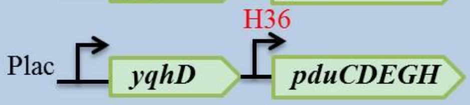
**PY03**	pEC-yqhD-glpF-H36-pdu	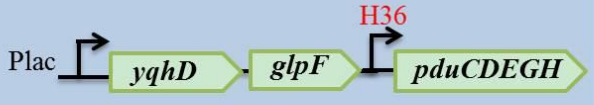
